# Psychosocial risks and the occurrence of work-related accidents

**DOI:** 10.1192/j.eurpsy.2021.1046

**Published:** 2021-08-13

**Authors:** N. Rmadi, N. Kotti, R. Masmoudi, F. Dhouib, K. Loukil, K. Jmal Hammami, M. Larbi Masmoudi, J. Masmoudi, M. Hajjeji

**Affiliations:** 1 Department Of Occupational Medicine, HEDI CHAKER hospital, SFAX, Tunisia; 2 Psychiatrie “a” Department, Hedi Chaker Hospital University -Sfax - Tunisia, sfax, Tunisia

**Keywords:** Psychosocial Risks, Occupational Accidents

## Abstract

**Introduction:**

Psychosocial risks (PSR) represent a new scourge of risks at work. The direct links between these risks and occupational accidents (OA) are not well documented, but some work restraints such as time pressure are common factors for both stress and accidents.

**Objectives:**

To establish a relationship between different PSR perceived by health staff and the occurrence of OA.

**Methods:**

Cross-sectional study conducted among staff working at Habib Bourguiba Hospital in Sfax from 1st January to 31 March 2015. The evaluation of mental health was performed by using the validated French version of questionnaire KARASEK.

**Results:**

The study involved 326 care staff (115 men and 211 women). The average age was 36 years old. The participants were mainly nurses (30.6%) and trainee physicians (35.6%). Blood exposure accidents were predominant (66.1% of cases) and were associated with high psychological demands at work with OR = 2.539 (95% CI [1.037 - 6.219]). Health care workers had a high psychological demand in 85.3% and a low latitude in 78.8% of cases. According to the Karasek model, tense employees accounted for 68.7% and assets 16.6%. OAs occurring during care were associated with night work and working in the emergency and resuscitation department (OR = 5,772 (95% CI [1,227-27,146] and OR = 5,778 (95% CI [1,702 -19,619]) respectively).

**Conclusions:**

The prevention of OA goes through the management of PSR, which remains a major concern for health and safety workers at work via the application of preventive strategies based on in-depth analysis of work situations.

